# Longitudinal associations between the frequency of playing Mahjong and cognitive functioning among older people in China: evidence from CLHLS, 2008–2018

**DOI:** 10.3389/fpubh.2024.1352433

**Published:** 2024-03-14

**Authors:** Lan Zhu, Yixi Wang, Yuju Wu, Amanda Wilson, Huan Zhou, Ningxiu Li, Yuanyuan Wang

**Affiliations:** ^1^West China School of Public Health and West China Fourth Hospital, Sichuan University, Chengdu, China; ^2^Faculty of Health and Life Sciences, De Montfort University, Leicester, United Kingdom; ^3^Key Laboratory of Brain, Cognition and Education Sciences, Ministry of Education, Guangzhou, China; ^4^Guangdong Key Laboratory of Mental Health and Cognitive Science, Center for Studies of Psychological Application, School of Psychology, South China Normal University, Guangzhou, China

**Keywords:** aging, Mahjong, cognitive function, cognitive ability, longitudinal study, CLHLS

## Abstract

**Background:**

Cognitive decline is prevalent among older adults, often resulting in decreased capabilities for self-care and a diminished quality of life. Mahjong, a culturally cherished and extensively played intellectual game in China, demands considerable cognitive function. While the cognitive benefits of playing Mahjong have been widely accepted, this study investigates an under explored aspect and aimed to ascertain the game’s potential contributions toward bolstering self-care abilities, enhancing overall quality of life, and mitigating against rising societal healthcare costs.

**Methods:**

The data analyzed in the study is collected from the Chinese Longitudinal Healthy Longevity Survey (CLHLS) with cognitive functioning being assessed through the Mini-Mental State Examination (MMSE). The frequency of playing Mahjong was measured through a self-reported questionnaire. Multiple linear regression models, latent variable growth models, and cross-lagged models were used to investigate the longitudinal relationship between game frequency and cognitive function in older people.

**Results:**

Of the 7,535 participants, the mean (SD) age was 81.96 (10.53) years. There were 7,308 (97%), 4,453 (59%), and 1,974 (26%) participants in 2011, 2014, and 2018, respectively. The results showed that Mahjong players had significantly higher MMSE scores compared to non-players from 2008 to 2018 (*β* = 0.893; *p* < 0.001), and non-players had significantly lower scores in 2011, 2014, and 2018 than in 2008 (*β* = −1.326, −0.912, −0.833; *Ps* > 0.05). Moreover, the frequency of playing Mahjong was associated with improved various cognitive domains. The declining frequency of playing Mahjong was substantially associated with the declining rate of MMSE scores (*r* = 0.336; *p* < 0.001). Mahjong frequency showed positive effects on MMSE scores, while the influence of Mahjong on MMSE scores were not significant.

**Conclusion:**

Playing Mahjong has a positive influence on the cognitive functioning among older people. It can help buffer against the decline in cognitive function and maintain cognitive function levels. The higher frequency of playing Mahjong is associated with improved reaction, attention and calculation, and self-coordination. A decline in the frequency of playing Mahjong was associated with a declining rate of cognitive function. The higher frequency of playing Mahjong among older people unilaterally influenced the improvement of cognitive function levels in older people in China.

## 1 Introduction

Negative impacts related to an aging population are considered a significant public health concern globally (1). By 2050, the population aged 65 and above is predicted to reach 20 percent worldwide (2). Furthermore, the population aged 80 or above is predicted to triple in number from 143 million in 2019 to an estimated 426 million by 2050 (3). The escalating aging population is predicted to result in larger economic burden for society (4). The prominent concern is that older people would experience cognitive impairments due to physical aging, which can render them incapable of basic self-care (5). Against the already strained social systems, governments, and the increasing role of society’s responsibility for care over familial care, increased utilization of social resources, the costs associated with providing fundamental care will inevitably increase (6). This strain is related to a large increase in older adults and the continuous decline of fertility rates in China during the last few years (7), with no indication that the proportion of older people in China over the next decade will decline, further exacerbating health and well-being issues among the aging population. The diminishing fertility rate also suggests that the number of carers for the increasing older population would be insufficient. Compared to adults, older people generally experience poorer health outcomes due to aging and various chronic diseases (8). They generally have poorer economic resources (9), are often without nearby family support (10), and thus require more care (11). In the current rapidly aging societal context, if older people cannot achieve better self-care, their physical health will further deteriorate, making it challenging to ensure a good quality of life.

Cognitive ability is the fundamental capacity of individuals to perceive (12), judge (13), recall (14), and control behavior (15) in response to information. Cognitive ability not only forms the basis for the self-care capabilities of older people, it is also a crucial skill for enhancing their quality of life (16, 17) and overall well-being (18, 19). However, it is widely accepted that cognitive decline is inevitable with aging (20). Therefore, methods to mitigate or delay the age-related cognitive decline in older people are important for successful self-care and for China to effectively address depleting social resource due to an aging population. Previous studies suggest that the medication to restore cognitive function for older people is not only costly (21) resulting in heavier financial burden (22), but works slowly and ineffectively in terms of cognitive improvement (23). Furthermore, medication may result in physical harm (24), leading to poor acceptance rates and adherence among older people (25). Therefore, it is crucial to pinpoint a non-pharmaceutical intervention that is suitable, low-cost, easily accepted from a cultural point of view, and is a sustainable method in order for Chinese older people to delay cognitive decline, restore basic cognitive functions, and promote long term engagement for successful self-care.

Mahjong is an important and widely popular intellectual game among Chinese people (26–28). It is a game that demands high cognitive functions (26). During Mahjong, players need to predict and assess their own and other participants’ moves using cognitive abilities such as visual perception (27), language comprehension (28), memory (28), and judgment to win (28). Mahjong typically consists of 136–152 tiles and is usually played by four individuals. To succeed, players must quickly identify their received tiles, memorize the moves of the other participants, and predict their next moves in order to adjust their own strategies and maximize their chances of winning (26). The process of playing Mahjong is not only an experience of mental and manual dexterity, but also requires quick reactions and focus, involving rapid coordination and cooperation (26). The process entails a series of cognitive processing steps, including attention, memory, and logical understanding (29). As a cognitive activity, Mahjong’s relationship with the cognitive functioning of older people requires further research. While there have been studies discussing the relationship between Mahjong and cognitive function level, revealing the positive influences of Mahjong on cognitive function level (26, 28), few studies have elucidated the factors influencing this positive effect. There is also limited research exploring the relationship between the frequency of playing Mahjong and cognitive abilities. Furthermore, few studies explore the relationship between Mahjong and cognitive functioning, those that do are predominantly cross-sectional, lacking longitudinal studies with long-term tracking of data to provide more accurate results regarding the relationship between Mahjong and cognitive function over time.

Employing multiple linear regression, this study aimed to investigate the relationship between playing Mahjong and cognitive function scores, further exploring the specific cognitive abilities influenced by Mahjong. Studies investigating the long-term covariation between the frequency of playing Mahjong and cognitive function in older people, using longitudinal data are scarce, with a lack of research explaining the sustained relationship between the frequency of playing and cognitive function. Subsequently, the study objectives were to use latent growth models to assess the long-term interactive relationship between the frequency of playing Mahjong and cognitive function scores. Finally, using cross-lagged models, the second objective was to identify the specific characteristics of this long-term interactive relationship. We hypothesized there would be an association between playing Mahjong and the cognitive function of older people, where a positive effect on cognitive function would be observed due to the higher frequency of playing Mahjong.

## 2 Materials and methods

### 2.1 Study design, participants, and data collection

This study employed data from the Chinese Longitudinal Healthy Longevity Survey (CLHLS), in a joint endeavor by the Center for Healthy Aging and Development Studies at Peking University and Duke University. The primary objective of the CLHLS is to comprehend various health aspects and their associated social, behavioral, and biological factors among China’s older people. The CLHLS survey commenced with an initial baseline assessment in 1998, followed by seven consecutive survey rounds conducted in 2000, 2002, 2005, 2008, 2011, 2014, and 2018. These surveys covered 22 sample locations across 31 administrative provinces, representing approximately 85% of the national population and spanning the eastern, western, and northeastern regions. Approximately half of the counties in the 22 provinces were randomly chosen as the primary survey units for the study.

We analyzed data from four CLHLS survey waves conducted from 2008 to 2018. To address the gaps in previous research and enhance the explanation of findings from prior studies, we selected data from four waves (2008, 2011, 2014, and 2018) from the nationally representative Chinese Longitudinal Healthy Longevity Survey (CLHLS). Among the four waves utilized 2008, acted as the baseline, with the baseline population tracked until 2018. The 2018 data represents the latest information in this database, ensuring the timeliness of our research conclusions.

**Figure 1:**
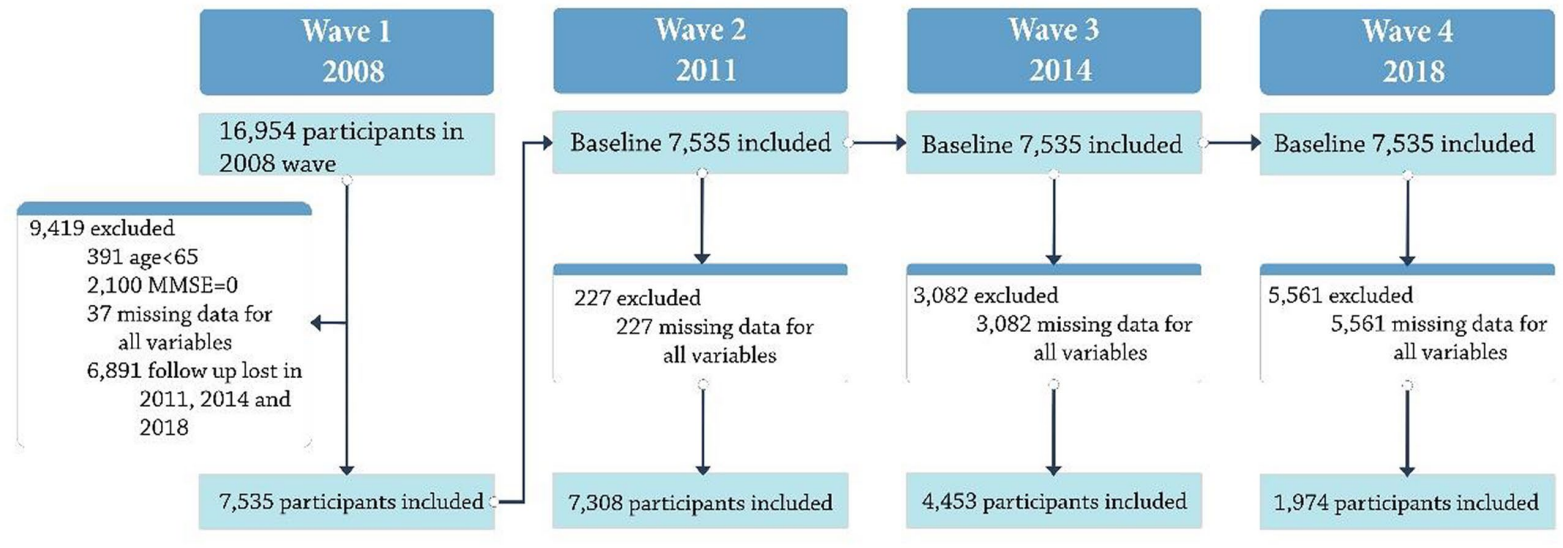
Flow chart of the study population.

Mortality rates and natural loss of contact with the respondents affected the attrition rate in the CLHLS data set (30). The main targets of the CLHLS survey are older people aged 65 and above, which is a group with a high mortality rate. Attrition rates for the four rounds of sampling from 2008 to 2018 in this study were 50.4, 37.7, and 53.5%, respectively. In each follow-up wave from 2008 to 2018, the number of participating CLHLS participants aged 64 or older gradually decreased due to dropouts and deaths. The total number of participants stood at 16,954 in 2008. Identity codes (ID) were employed as the solitary matching criterion for longitudinally integrating data across the four waves. After filtering out participants aged below 65, those with 2008 MMSE scores equal to zero, missing data entries, participants who only participated at baseline and lacked follow-up in 2011, 2014, and 2018. There were 7,535 participants in the initial cohort (round 1), who were reassessed in 2011 (round 2), 2014 (round3), and 2018 (round 4). There were 7,308 s round participants (97%), 4,453 third-round participants (59%), and 1,974 participants in the fourth-round (26%). Figure 1 depicts the process of sample screening.

### 2.2 Variables

#### 2.2.1 Cognitive function

The Mini-Mental State Examination (MMSE), also known as the Mini-Mental State Exam or the Simple Mental State Examination, was developed by Folstein et al. in 1975 (31). The questionnaire encompasses an evaluation of five dimensions of cognitive function: general ability, reaction ability, attention and calculation abilities, recall capability, and self-coordination ability (32). It stands as one of the most influential standardized tools for assessing intellectual status. Participants of the CLHLS all underwent a cognitive assessment using the Chinese version of the MMSE questionnaire, widely used in academic (33, 34) and clinical settings. Validated from previous studies involving Chinese older people, this version incorporates cultural and socioeconomic factors to retain its applicability and relevance (35). The MMSE score spectrum ranges from 0 to 30, a total score above 24 on the scale indicates normal cognitive function, with a higher score denoting superior cognitive performance (36). A total score below 24 suggests cognitive impairment (36). The Cronbach’s alpha coefficients for the rounds in 2008, 2011, 2014, and 2018 were 0.92, 0.96, 0.96, and 0.96, respectively.

#### 2.2.2 The frequency of playing Mahjong

At the onset participants of CLHLS were asked, “Do you play Mahjong at present?” to rate their frequency of playing Mahjong. Answers consisted of “almost every day,” “once a week, but not daily,” “at least once a month, but not weekly,” “sometimes, but not monthly” and “never.” For analysis, the frequency ranges from 0–2 points: 0 denotes never playing Mahjong; 1 denotes occasional play (combining “once a week, but not daily,” “at least once a month, but not weekly,” and “sometimes, but not monthly”); 2 denotes playing Mahjong daily.

### 2.3 Control variables

The relationship between the frequency of playing Mahjong and cognitive function may be influenced or confounded by other factors. To control for these potential confounding factors, we selected covariates related to the frequency of playing Mahjong and cognitive function. Based on available data, the chosen control variables for this study included age (65–79, 80–89, 90–99, over 100), sex (male, female), marital status (living with/ without spouse), schooling years (No schooling, 1–6 years, and over 7 years), residence (urban, rural), financial support (sufficient/insufficient financial support), smoking status (smoking, no smoking) and alcohol consumption (drinking, no drinking). Among them, age (37), sex (38), marital status (39), schooling years (40), residence (30), and financial support (41) were common socio-demographic variables, which can reflect the basic characteristics and social background of older people, and are also important factors affecting the frequency of playing Mahjong and cognitive function. Smoking status (42) and alcohol consumption (43) serve as indicators of healthy habits, potentially impacting the risk of cardiovascular and cerebrovascular diseases, thereby affecting the cognitive function and the frequency of playing Mahjong among older people. Further details can be found in Table 1.

**Table 1:** Demographic characteristic of the frequency of playing Mahjong, and MMSE score of the study population at 2008 (baseline), 2011 (3-year), 2014 (3-year), 2018 (4-year) follow-up.

Characteristic	Wave 2008 (*n* = 7,535)	Wave 2011 (*n* = 7,308)	Wave 2014 (*n* = 4,453)	Wave 2018 (*n* = 1,974)
	Mean or %	Mean or %	Mean or %	Mean or %
Age (65–117), *M* (SD)	81.96 (10.53)	85.07 (10.52)	84.68 (9.53)	85.03 (7.79)
**Age group, *n* (%)**				
65–79	3,218 (42.71)	2,533 (34.66)	1,607 (36.09)	576 (29.18)
80–89	2,325 (30.86)	2,175 (29.76)	1,510 (33.91)	862 (43.67)
90–99	1,425 (18.91)	1,858 (25.42)	1,006 (22.59)	438 (22.19)
≥100	567 (7.52)	742 (10.15)	330 (7.41)	98 (4.96)
**Sex, *n* (%)**				
Male	3,493 (46.36)	3,384 (46.31)	2,103 (47.23)	916 (46.4)
Female	4,042 (53.64)	3,924 (53.69)	2,350 (52.77)	1,058 (53.6)
**Marital status, *n* (%)**				
Living with spouse	3,240 (43)	2,764 (37.82)	1,791 (40.22)	799 (40.48)
Living without spouse	4,295 (57)	4,544 (62.18)	2,662 (59.78)	1,175 (59.52)
Schooling years (0–24), *M* (SD)	2.44 (3.6)	2.42 (3.59)	2.6 (3.6)	2.82 (3.69)
**Schooling years, *n* (%)**				
No schooling	4,184 (55.53)	4,067 (55.65)	2,326 (52.23)	961 (48.68)
1–6 year+ schooling	2,461 (32.66)	2,389 (32.69)	1,557 (34.97)	744 (37.69)
7 year+ schooling	890 (11.81)	852 (11.66)	570 (12.8)	269 (13.63)
**Residence, *n* (%)**				
Urban	1,325 (17.58)	1,472 (20.14)	832 (18.68)	357 (18.09)
Rural	6,210 (82.42)	5,836 (79.86)	3,621 (81.32)	1,617 (81.91)
**Financial support, *n* (%)**				
Sufficient financial support	5,857 (77.73)	5,724 (78.33)	3,589 (80.6)	1,699 (86.07)
Insufficient financial support	1,678 (22.27)	1,584 (21.67)	864 (19.4)	275 (13.93)
**Smoking status, *n* (%)**				
Smoking	1,576 (20.92)	1,363 (18.65)	797 (17.9)	314 (15.91)
No smoking	5,959 (79.08)	5,945 (81.35)	3,656 (82.1)	1,660 (84.09)
**Alcohol consumption, *n* (%)**				
Drinking	1,505 (19.97)	1,289 (17.64)	696 (15.63)	293 (14.84)
No drinking	6,030 (80.03)	6,019 (82.36)	3,757 (84.37)	1,681 (85.16)
Play mahjong (0–2), *M* (SD)	0.27 (0.59)	0.23 (0.55)	0.25 (0.57)	0.22 (0.56)
**Play mahjong group, *n* (%)**				
Never	6,062 (80.45)	6,116 (83.69)	3,664 (82.28)	1,668 (84.5)
Sometimes	916 (12.16)	715 (9.78)	476 (10.69)	168 (8.51)
Almost everyday	557 (7.39)	477 (6.53)	313 (7.03)	138 (6.99)
MMSE (0–30), *M* (SD)	25.28 (5.68)	23.20 (8.37)	23.82 (8.06)	23.93 (7.98)
General Ability (0–12)	10.80 (2.14)	9.94 (3.34)	10.12 (3.21)	10.16 (3.16)
Reaction Ability (0–3)	2.57 (0.91)	2.37 (1.12)	2.49 (1.03)	2.52 (1.02)
Attention and Calculation Abilities (0–6)	4.29 (1.90)	3.84 (2.26)	4.01 (2.20)	3.99 (2.21)
Recall capability (0–3)	2.20 (1.16)	1.98 (1.27)	2.09 (1.23)	2.08 (1.24)
Self-Coordination Ability (0–6)	5.41 (1.24)	5.07 (1.81)	5.11 (1.77)	5.18 (1.73)
**Cognitive impairment, *n* (%)**				
No	5,575 (73.99)	4,887 (66.87)	3,154 (70.83)	1,400 (70.92)
Yes	1,960 (26.01)	2,421 (33.13)	1,299 (29.17)	574 (29.08)

## 3 Method

Multiple linear regression was employed to explore the effect of Mahjong on cognitive function in older people and analyzed the ability of Mahjong against specific cognitive abilities. Then the latent growth models were used to separately estimate the trajectories of MMSE scores and the frequency of playing Mahjong, analyzing the longitudinal correlation. Latent growth modeling is a common model used in longitudinal data analysis, which aims to explore the process of variable change and development over a specified time period (44). It is not only concerned with the developmental trend of a certain traits over time, but also with the individual differences in that trend (45). Common factors that affect the decline in cognitive function in older people (46), such as age (47), sex (48), educational background (40), smoking status (49), and alcohol consumption (50) are controlled for. The latent growth modeling method therefore can be used to explore the changing trends and rates of the frequency of playing Mahjong and cognitive scores over time, further specifying the covariant relationship between them. This approach can ensure more accurate analysis of the relationship between the frequency of playing Mahjong and cognitive function as well.

Finally, cross-lagged models were employed to explore the longitudinal reciprocal association between the frequency of playing Mahjong and cognitive function between 2008 and 2018. Cross-lagged modeling is a longitudinal method of data analysis that describes the interrelationships among the variables (51). Utilizing long-term tracking data for longitudinal studies can not only enhance our understanding of the relationship between Mahjong and cognitive function, but it can as elucidate how this relationship evolves over time. The extended tracking in longitudinal studies provide more participant-related control variables that mitigate errors arising from individual differences among participants, they also control for participant-specific factors influencing the study outcomes. This approach enables a more accurate assessment of how Mahjong influences cognitive function over time, thereby enhancing the accuracy and reliability of research outcomes. Moreover, employing a cross-lagged model can help explore the causal relationship between the frequency of playing Mahjong and cognitive function levels.

### 3.1 Multiple linear regression

#### 3.1.1 The relationship between Mahjong games and cognitive function

To investigate the impact of playing Mahjong on the cognitive function of older people, two multiple linear regression models were employed, controlling for all covariates (including age, sex, marital status, schooling years, residence, financial support, smoking status, and alcohol consumption). The MMSE score was the dependent variable and the data was measured over four time points (2008, 2011, 2014, and 2018) as independent variables in two groups (Mahjong players and non-players) to test changing trends of the MMSE total scores. To test the different trend between the two groups, multiple linear regression modeling was also employed with the two groups (Mahjong playing, non-Mahjong playing) over the 4 time points (2008, 2011, 2014, and 2018) as independent variables, while MMSE total scores were entered as the dependent variable.

#### 3.1.2 The relationship between the frequency of Mahjong and cognitive functions

To further analyze the specific dimensions of cognitive function improvement in older people resulting from playing Mahjong, five cognitive function dimensions of the MMSE scale (general ability, reaction, attention and calculation, recall, and self-coordination abilities) were included. These five dimensions were then combined with multiple linear regression to examine the effects of the frequency of playing Mahjong on enhancing specific cognitive abilities of older people, over the same time points mentioned above. Next, two general linear regression models were run, with the MMSE total score as the dependent variable across the four time points (2008, 2011, 2014, and 2018), while Mahjong participation (players vs. non-players) served as the independent variable. This was to test changing trends of the five dimensions of MMSE scores. General linear regression modeling, controlling for all covariates, set within the 3 groups (never play Mahjong, sometimes play Mahjong, and play Mahjong almost every day) over the 4 time periods (2008, 2011, 2014, and 2018), functioned as independent variables. The five dimensions of MMSE scores functioned as dependent variables to assess the differences between the three groups and identify changing trends among the MMSE total scores.

### 3.2 Latent growth models

**Figure 2:**
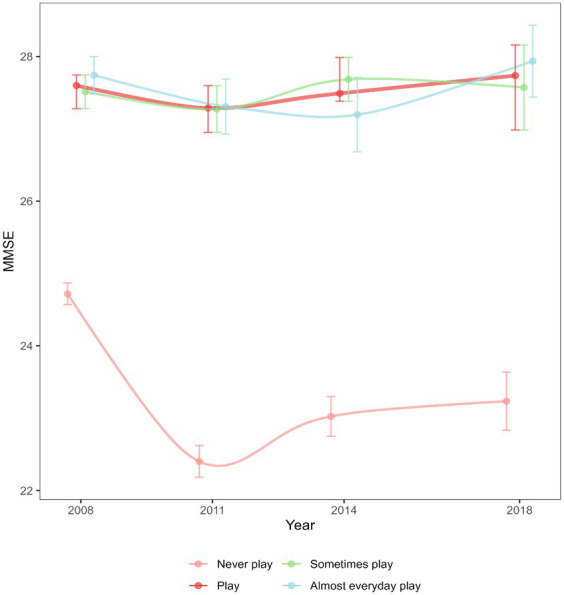
The MMSE scores in older people from 2008 to 2018.

Initially, two linear latent growth models with incorporated random effects were developed for the MMSE scores and the frequency of Mahjong, allowing for individual trajectory variances. These models included time-bound intercepts and slopes, effectively representing the trajectories of MMSE scores and Mahjong frequency. Each model captured the average baseline level (intercept), the average change over 3 years (slope) from baseline to the most recent assessment, differences among individuals at baseline, and variations among individuals over time. Subsequently, the models for the frequency of playing Mahjong and MMSE scores were integrated into a single model. Simultaneous estimates were carried out to establish their relationship, as depicted in Figure 2. The estimated parameters encompass correlations between the baseline values, baseline values and the slopes, and within the slope correlations. The equation for the model incorporated control variables, including those that remain constant over time such as sex, and schooling years, as well as control variables that change over time, including marital status, residence, financial support, smoking status, and alcohol consumption.

### 3.3 Cross-lagged models

**Figure 3:**
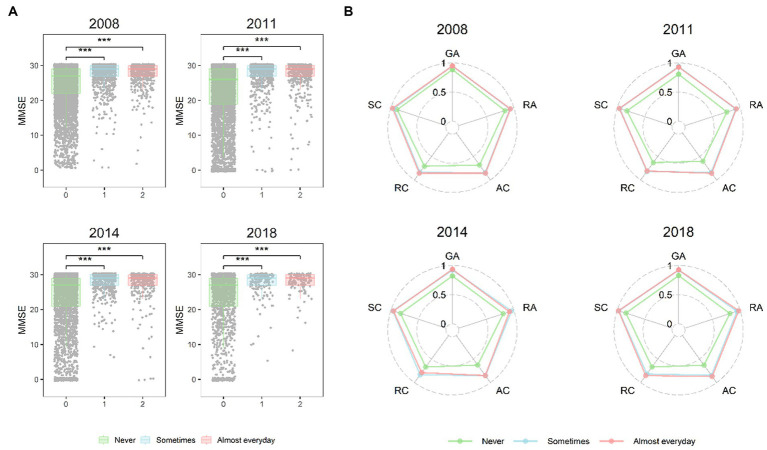
**(A)** The box plots of MMSE scores of play Mahjong groups in older people from 2008 to 2018, **(B)** five dimensions of MMSE scores in older people from 2008 to 2018. GA, General Ability; RA, Reaction Ability; AC, Attention and Calculation Ability; RC, Recall Ability; SC, Self Coordination Ability.

The latent growth model cannot establish whether the link between changes in the frequency of Mahjong and the MMSE score were a result of the frequency Mahjong influencing MMSE scores or vice versa. Cross-lagged modeling is a longitudinal data analysis method that describes interrelationships between variables (51). It can reveal longitudinal reciprocal relationships among different variables at multiple time points (52) and is mainly used to longitudinal reciprocal clarify the causal relationships. Cross-lagged modeling controls for autoregressive effects, ensuring the stability of variables across different periods, and provides better reflection of dynamic relationships between variables rather than static relationships. This approach helps avoid drawing erroneous conclusions due to short-term fluctuations. The cross-lagged model analyzes the ability of one variable’s past to predict the future values of another variable, along with the direction of the prediction (53). Using cross-lagged models, we studied the longitudinal reciprocal connection between the frequency of playing Mahjong and MMSE scores. The model evaluated the relationship between MMSE scores and the frequency after controlling for their stability via an autoregressive route (i.e., after accounting for the prior level of MMSE scores and the frequency of playing Mahjong). In Figure 3, the regression of the frequency at each period was based on the frequency (at the prior time) and the MMSE score (at the prior time). Similarly, the regression of the MMSE score, at each time point, was based on the prior MMSE score and the frequency. The correlation between the frequency of playing Mahjong and the MMSE score measured at the same time was permitted.

To construct a straightforward cross-lagged model and calculate the average effects across the periods, we standardized coefficients possessing similar meanings across three distinct time spans, covariates (age, sex, marital status, schooling years, residence, financial support, smoking status, alcohol consumption) acting as the controlling confounding factors. Figure 3 illustrates the model constraints, with uniform restricted paths indicated by consistent colors and identical letters in the illustrating drawing. Model fit statistics, including the CFI (Comparative Fit Index) and TLI (Tucker-Lewis Index), were employed to evaluate all models. CFI and TLI values exceeding 0.90 suggest a robust fit (54). Furthermore, the RMSEA (root mean square error of approximation) was also leveraged and values below 0.08 reflected a satisfactory model fit (54).

All study participants, irrespective of missing items, were included in the analysis. In cases of incomplete data, latent growth models and cross-lagged models employed the Maximum Likelihood method to estimate missing data of MMSE scores and the frequency of playing Mahjong. Multiple imputations were employed to fill in missing values of covariates. Analytical procedures were executed utilizing R (version 4.2.1) and Mplus 8.0 software packages (Muthén & Muthén) (55).

## 4 Results

### 4.1 Descriptive statistics of participants in China

At baseline, the study comprised 7,535 participants, including 4,042 (53.64%) females (Table 1). There were 7,308 (97%), 4,453 (59%), and 1.974 (26%) participants in the 2011, 2014, and 2018 of studies, respectively. The mean (SD) of MMSE scores was 25.28 (5.68) at the 2008 (baseline), and 23.20 (8.37), 23.82 (8.06), and 23.93 (7.98) in the 2011 (second), 2014 (third), and 2018 (fourth) rounds.

### 4.2 The result of multiple linear regression

#### 4.2.1 The relationship between Mahjong games and cognitive function in older people from 2008 to 2018

Form the multiple linear regression models, the results revealed that older people who played Mahjong exhibited significantly higher MMSE total scores compared to non-players (*β* = 0.893; *p* < 0.001) (Figure 2). Older people who played Mahjong sometimes and almost every day had significantly higher MMSE total scores compared to non-players in 2008, 2011, 2014 and 2018 (*Ps* < 0.001) respectively (Figure 3B).

The MMSE scores of older people exhibited an interaction effect in the grouping (playing Mahjong or not) over time (2008, 2011, 2014, 2018) (*β* = 1.716, 1.465, 1.973; *Ps* < 0.001). Among the older people who play Mahjong, there was no significant difference in MMSE scores between 2008, 2011, 2014, and 2018 (*Ps* > 0.05). However, non-players’ MMSE scores in 2011, 2014, and 2018 were significantly lower than the scores in 2008 (*β* = −1.326, −0.912, −0.833; *Ps* < 0.001).

#### 4.2.2 The relationship between the frequency of Mahjong and specific cognitive functions in older people from 2008 to 2018

We analyzed the distinctive characteristics of Mahjong in enhancing the specific dimensions of cognitive function of older people from 2008 to 2018 (Figure 3B). The results revealed that compared with non-players in 2008, the frequency of playing Mahjong was associated with enhancements in reaction time, attention and calculation, recall capability, and self-coordination abilities. Older people who play Mahjong occasionally in 2011 and 2014 (*β* = 0.062, 0.055, *Ps* < 0.01) and those who played Mahjong daily in 2011 and 2018 (*β* = 0.056, 0.067, *Ps* < 0.05) had higher scores in reaction ability. Older people who played Mahjong occasionally and daily had higher scores in attention and calculation, recall, and self-coordination abilities in 2011, 2014 and 2018 (*P*s < 0.01).

### 4.3 The result of latent growth models

**Figure 4:**
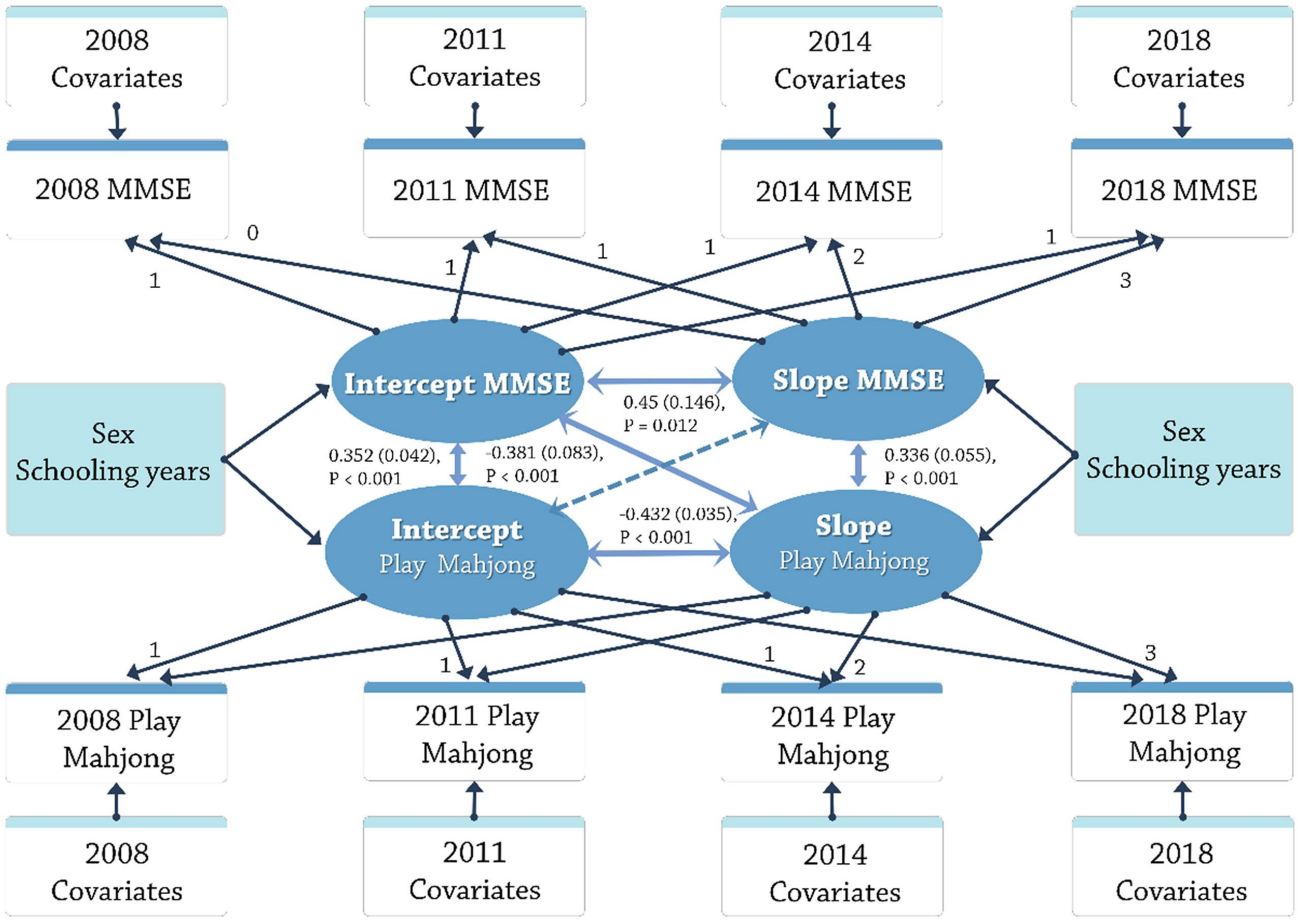
Latent growth model of the frequency of play mahjong and MMSE.

The initial average frequency (intercept) of playing Mahjong for this sample was 0.265 (*SE*: 0.006; *p* < 0.001), indicating the initial frequency was lower than that of occasional players (lower frequency). The baseline average MMSE score (intercept) of 25.262 (*SE* = 0.063; *p* < 0.001) demonstrated that this sample initially exhibited average cognitive functioning (MMSE score ≥ 24). The linear latent growth models indicated that both frequency (slope was −0.028; *p* < 0.001) and MMSE scores (slope was −1.778; *p* < 0.001) deceased, respectively, over the time period. Significant individual differences were found in both trajectories for the variances of intercept and slope for MMSE scores and the frequency of Mahjong. Figure 4 illustrates simultaneous equations showing paths of statistical significance (marked by solid lines) and paths that lack statistical significance (represented by dashed lines). The simultaneous equation model demonstrated superior fit with the data (CFI: 0.975; TLI: 0.966; RMSEA: 0.020). Positive correlations were found between the initial intercept (*r* = 0.352; *SE* = 0.042; *p* < 0.001) and slope (*r* = 0.336; *SE* = 0.055; *p* < 0.001) of the frequency of playing Mahjong and the MMSE scores, suggesting a cross-sectional and longitudinally correlation between the frequency and cognitive decline.

### 4.4 The result of cross-lagged models

**Figure 5:**
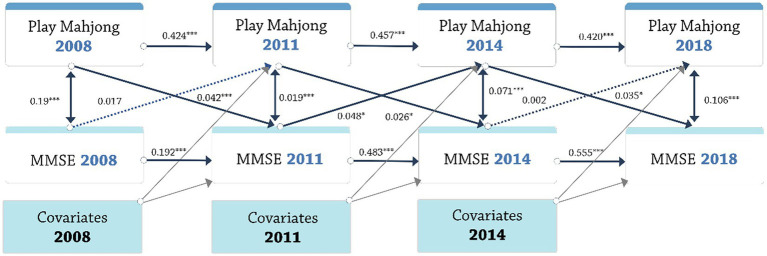
Cross-lagged model of the frequency of play mahjong and MMSE.

The final cross-lagged model fit the data excellently, as evidenced by the CFI (0.957), TLI (0.953), and RMSEA (0.016). When analyzing the standardized coefficients, the influence of the frequency of playing Mahjong on MMSE scores showed a positive influence from 2008 to 2011, 2011–2014, and 2014–2018 (standardized *β* = 0.042, 0.026, 0.035; *Ps* < 0.05). From 2008 to 2011 and 2014–2018, the influence of MMSE scores on the frequency of playing Mahjong was not significant (standardized *β* = 0.017, 0.002; *Ps* > 0.05) while from 2011 to 2014 a positive influence was significant (standardized *β* = 0.048; *p* < 0.05). The result showed that the increase in the frequency of playing Mahjong contribute to the improvement of the MMSE scores of older people, while the higher the MMSE scores may not relate to higher frequency of playing Mahjong (Figure 5).

## 5 Discussion

To the authors’ knowledge this is the first longitudinal study examining the long-term covariant characteristics between playing Mahjong and cognitive function in older people. The results substantiated our research hypothesis, providing evidence for the positive influence of Mahjong on the cognitive function of older people. Importantly, through latent growth models and cross-lagged models, the research identified a prolonged, stable, unidirectional predictive relationship between the frequency of playing Mahjong and cognitive function, which had not been investigated within the existing literature.

We found that playing Mahjong can help slow down the decline in cognitive function in older people over time, maintaining their cognitive function levels. Among the older people who played Mahjong, there was no significant difference in MMSE scores between 2008, 2011, 2014, and 2018. However, among those who did not play Mahjong, MMSE scores in 2011, 2014, and 2018 were significantly lower than the scores in 2008. As individuals age, there is a rapid decline in cognitive function among older people, particularly for those considered to have advanced age (56–59). Numerous research findings indicate a sharp downward trend in cognitive function as age increases (5, 60–62). In our study, older people who did not play Mahjong experienced a significant and unrecovered decline in cognitive function with age, aligning with a substantial body of previous research (26, 63, 64). However, the rate of decline in cognitive function with age was slower for older people playing Mahjong. In comparison to 2008, the cognitive function of older Mahjong players remained relatively stable. Moreover, the cognitive function of older Mahjong players in 2018 was even greater than that in 2008. This suggests that playing Mahjong can effectively alleviate age-related decline of cognitive function or even improve the function among older people in China.

To further analyze the positive effect of specific cognitive abilities, we differentiated cognitive abilities based on the five cognitive function dimensions of the MMSE scale (general ability, reaction, attention, and calculation, recall, and self-coordination abilities). The results from 2008 to 2018 revealed that the frequency of playing Mahjong can enhance reaction, attention and calculation, recall, and self-coordination abilities. Mahjong, like other cognitive games, demands continuous logical reasoning during gameplay (65). Players must analyze and strategize with the titles they receive, then engage in constant logical operations (66). Furthermore, the game requires participants to memorize the titles discarded by others in each round, contributing to memory exercises (67). Prior research indicates that strategic games can enhance individuals’ logical reasoning skills (29). Specifically, games like Mahjong, are considered to improve memory accuracy and reaction ability (29, 67). Our research findings align with these previous studies (29, 32–35). Moving to the data from 2011, older people who played Mahjong not only exhibited higher scores in attention and calculation but also demonstrated higher scores in language, understanding, and self-coordination. This suggests that games that build upon improvements in attention and calculation like Mahjong have an additional positive buffer against language-related cognitive function decline. As Mahjong is a multiplayer game involving verbal communication and strategic decision-making, it facilitates language exchange and understanding of social cues during gameplay. Previous research on gambling games has also explored their relationship with social interaction, indicating a reduction in anxiety scores among socially anxious individuals engaged in gambling (68). Moreover, the Social Engagement Theory suggests that social activities can rebuild the social networks of older people (69), enhance their social support (70), and consequently improving their cognitive abilities (71). Mahjong, as a social activity, suggests that participation can lead to improvements in the language and comprehension abilities of older people. And it involves the use of various social interaction skills, such as language communication and emotional expression (68). In other words, Mahjong, as a cognitive activity, not only positively influences older people’s reaction and memory-based abilities, but also enhances their language and self-coordination skills through social interaction during the game. Furthermore, the benefits of regular Mahjong activities on reaction, attention and calculation, and self-coordination suggests that the decline in these cognitive functions may be controllable (28), or even reversible (72). Older people can mitigate age-related cognitive decline and improve their reaction, sustain attention and calculation, enhance memory, and increase self-coordination through regular Mahjong activities. Moreover, the positive effects of Mahjong on these cognitive abilities imply their interrelatedness (73) and mutual reinforcement (67). Improved reaction can influence attention and calculation abilities, leading to the maintenance and restoration of memory in older people, ultimately facilitating better self-coordination. Previous research has also found the beneficial effects of attention and calculation abilities on memory (74, 75), with individuals demonstrating higher levels of attention and calculation abilities also exhibiting stronger memory (76, 77). Additionally, several cross-sectional studies have identified a positive correlation between cognitive abilities and self-coordination (78–80), indicating that higher cognitive levels are associated with higher levels of self-coordination. Our study supplements and supports these potential underlying mechanisms among cognitive abilities over time, elucidating the specific positive effects of Mahjong on the temporal changes in older people’s cognitions. However, the sequential relationships between these cognitive abilities over time require further exploration.

In the latent variable growth model, the result shows a positive correlation between the declining rate in frequency of playing Mahjong and the declining rate in cognitive scores among older people. Over the period from 2008 to 2018, the decline in frequency was positively correlated with the decline in cognitive scores among older people, indicating a covariant relationship between low frequency of playing Mahjong and deteriorating cognitive function. Previous studies have also identified a relationship between cognitive games and cognitive function levels (28, 72, 81). A study that used Cox proportional hazards models to investigate the risk of dementia among Chinese older people who play Mahjong, revealing a reduced risk (81). Other studies have found that older people who play card games, compared to those who do not, show a slower decline in cognitive abilities and lower risk of dementia (72). Our study suggests that playing Mahjong contributes to maintaining the cognitive levels of older people. Notably, the novel finding is that even after controlling for various factors that influence the decline in cognitive function in older people, the results still indicate a long-term covariant relationship between a decrease in Mahjong playing frequency and a decline in cognitive function in older people. Earlier studies often had fewer controls for other factors influencing cognitive decline in older people and primarily focused on the impact of playing/not playing Mahjong on cognitive function without discussing the long-term characteristics of the relationship. The discovery in this study suggests that over time a decrease in frequency of playing Mahjong is associated with a decline in cognitive function and that Mahjong has a significant and enduring role in maintaining cognitive function in older Chinese people. Earlier studies have already identified a positive relationship between increased frequency of playing Mahjong and enhanced cognitive function (26, 28). A study indicated that playing Mahjong could aid in the augmentation of cognitive capabilities in older people (67). Over the course of the12-week study, it was found that playing Mahjong three times weekly significantly advances the executive functions in older people with marginal cognitive impairment (72). Our research further supports this conclusion from the opposite perspective: the lower the frequency of playing Mahjong, the faster the decline in cognitive function.

Compared to previous research, our study not only confirms the stable long-term association between the frequency of playing Mahjong and cognitive function levels, but also clarifies the predictive capacity of the frequency in terms of changes in cognitive function levels over time. The results demonstrate that the frequency of playing Mahjong in 2008, 2011, and 2014 positively influenced cognitive function scores in 2011, 2014, and 2018. Conversely, cognitive function scores in 2008, 2011, and 2014 did not significantly influence the frequency of playing Mahjong in 2011, 2014, and 2018. This indicates a longitudinal unidirectional relationship between Mahjong frequency and cognitive function. In other words, higher frequency of playing Mahjong significantly improved cognitive function scores in the subsequent time periods, and this relationship was unidirectional. This conclusion, combined with the previous latent variable model results, further supports our hypothesis that regular Mahjong games had a positive influence on the cognitive abilities of older people. While previous studies have already identified the positive impact of Mahjong games on the cognitive functioning of the older people (26, 28), their lack of data tracking made their conclusions ungeneralizable over time and less stable. Moreover, our study specifies the unidirectional positive relationship between the frequency of playing Mahjong and cognitive function levels in the older people, further emphasizing the significance of Mahjong games in maintaining and enhancing the cognitive function. Therefore, Mahjong, a low-cost, minimally resource-intensive, and easily accessible cognitive game, compared to traditional monotonous cognitive training and cognitive rehabilitation exercises, requires almost no specialized guidance (82), supervision (83), or training (84). It thus has minimal constraints on the training environment. Through Mahjong games, cognitive decline in older people can slow, and cognitive function levels can be maintained. Moreover, with higher frequencies of playing Mahjong, cognitive function in the older people can even recover and improve. Additionally, no significant relationship was found between improved cognitive function and increased frequency of Mahjong games. Older people with higher cognitive levels may be interested in more challenging intellectual activities, and Mahjong games could be just one of those activities. As cognitive function is restored or maintained, older people may engage in a broader range of leisure and recreational activities.

Mahjong is a low-cost, socially accessible cognitive game that older people find easy to engage with and enjoy maintaining among social circles. As a method to alleviate or delay age-related cognitive decline, it plays a crucial role in effectively mitigating against the inadequate social medical insurance capacity and the scarcity of social medical resources as seen with the aging population in China. For older people, compared to medication therapy for cognitive function improvement, playing Mahjong incurs lower economic costs (67) and is safer for their physical well-being (29). Medication therapy often requires longer treatment periods (85), entails higher expenses (86), and may not yield satisfactory results (87), thus exacerbating the economic burden on older adults and posing challenges for long-term medication adherence (88). Moreover, medication therapy may adversely affect older people’s physical health (89), such as liver and kidney (90), damage gastrointestinal discomfort (91), and could lead to dependency and potential abuse (92), thereby jeopardizing their well-being. Lastly, Mahjong, being a multiplayer game, fosters social interaction among older adults, effectively alleviating loneliness (71) and depression (93), enhancing their quality of life and happiness (29), which medication therapy cannot achieve. Playing Mahjong can enhance various cognitive abilities in older people, such as attention, reaction, and calculation, thereby helping to prevent and delay the onset of dementia (94). Therefore, Mahjong requires minimal additional social investment, resulting in less pressure on social medical resources and alleviating the economic costs (95). Moreover, older people willingly participate and can afford to sustain this activity.

Mahjong games can also have a detrimental impact on older people. In some cases, Mahjong can become a form of gambling (96), leading to financial losses among older people (97). Excessive gambling can deplete savings and retirement funds (98), leading to financial instability and increased stress (99). Prolonged periods of sitting and intense concentration during Mahjong games may contribute to a sedentary lifestyle among older people, increasing their risk of health problems such as obesity (100), cardiovascular disease (101), and musculoskeletal issues (102). Older people who become overly preoccupied with Mahjong may neglect other important activities such as exercise (103), socializing with family and friends (98), and engaging in hobbies (104), which could lead to a decline in overall well-being and quality of life (105).

The positive effects of Mahjong that enhance cognitive function on the other hand contribute to improving quality of life, offering new perspectives for social care for older people. It is essential to promote balanced and responsible participation in Mahjong. Future research can further explore the neurological effects of Mahjong on the cognitive recovery among older people and its relationship with recovery time, thus providing a deeper understanding of cognitive decline in older people and better addressing the challenges posed by an increasingly aging population in China.

## 6 Study limitations

The research on the frequency of playing Mahjong among older people in this study may be subject to recall bias due to their age. Future research should focus on employing more robust methods to minimize the influence of recall bias and enhance the accuracy of study outcomes, even though the MMSE is a standardized test. Furthermore, our study primarily includes older people, whose cognitive decline is more severe and less reversible due to the aging process. However, studying an older population can contribute to a better understanding of the role of cognitive function maintenance and recovery when promoting self-care and enhancing quality of life, which addresses the challenges faced by an aging population allowing for evidence to be implemented to facilitate early interventions for cognitive decline.

In addition, the Mini-Mental State Examination (MMSE) scale has some limitations, which have implications for our findings. First, the MMSE may lack sensitivity in detecting mild cognitive impairment (MCI) (106) or early dementia (106), especially in highly educated populations (107) or in older populations with atypical manifestations of cognitive decline (108), which may affect the ability to capture subtle changes in cognitive impairment associated with activities such as playing Mahjong. Secondly, the MMSE primarily assesses orientation (86), attention (109), memory (109), and language (109), but is not designed to assess the performance function of behavior and executive function (106), cognitive domains that are important for complex activities such as Mahjong playing, which also involves strategic thinking (29) and decision-making (94). The MMSE score may not specifically reflect the cognitive abilities required to play Mahjong. While it provides a general assessment of cognitive functioning, it may not capture the subtle cognitive skills involved in Mahjong, such as pattern recognition (110), spatial reasoning (72), and attention to detail (111). Third, performance on the MMSE can be affected by factors such as education (106), cultural background (106), and language proficiency (106). Since Mahjong is a popular game with cultural significance in China, individuals familiar with the game may perform better on tasks associated with it (110), regardless of their overall cognitive functioning. This cultural factor may affect the interpretation of MMSE scores in the context of playing Mahjong games.

Finally, while this study explores the positive effects of playing Mahjong on specific cognitive functions, this exploration remains a cross-sectional comparison and cannot indicate whether the improvement in these specific cognitive function dimensions follows a certain order. This limitation arises primarily from a higher number of individuals lost to follow-up over time. Future research can address this limitation more comprehensively by exploring the potential sequence of cognitive function recovery.

## 7 Conclusion

Playing Mahjong is beneficial in slowing down cognitive decline in older people. The higher frequency of playing Mahjong is associated with improved reaction, attention and calculation, and self-coordination abilities. A decrease in Mahjong- frequency is associated with declining cognitive function both cross-sectionally and longitudinally over time. Higher frequency of playing Mahjong in older people unidirectionally influences the improvement of cognitive function levels. Our findings demonstrate the significant role of Mahjong games in slowing down the decline of cognitive abilities among older people. Promoting the playing of Mahjong and increasing its frequency was determined as mitigating against a decline in cognitive function and restore the cognitive functioning of older people.

## Data availability statement

The original contributions presented in the study are included in the article/Supplementary material, further inquiries can be directed to the corresponding author.

## Ethics statement

The studies involving human participants were reviewed and approved by Center for Healthy Aging and Development Studies at Peking University and Duke University. The studies were conducted in accordance with the local legislation and institutional requirements. The participants provided their written informed consent to participate in this study. YixW registered the platform and obtained permission to use the data.

## Author contributions

LZ: Conceptualization, Funding acquisition, Investigation, Methodology, Project administration, Supervision, Visualization, Writing – original draft, Writing – review & editing. YixW: Data curation, Formal analysis, Resources, Software, Validation, Visualization, Writing – original draft, Writing – review & editing. YujW: Investigation, Methodology, Writing – review & editing. AW: Formal analysis, Methodology, Software, Writing – review & editing. HZ: Software, Visualization, Writing – review & editing. NL: Software, Visualization, Writing – review & editing. YuaW: Writing – review & editing, Conceptualization, Methodology, Project administration, Supervision.
